# Supplementary material for: Divergent biological consequences of APOE isoforms across industrialized and non-industrial environments
Source: PLoS Genet. 2026 Sep 8;22(9):e1012285. doi: 10.1371/journal.pgen.1012285 (PMC13581214; doi:10.1371/journal.pgen.1012285)
Supplement: Supplementary file 1 [file pgen.1012285.s001.docx]

## Supplementary Materials for Watowich et al.

## “Divergent Biological Consequences of APOE Isoforms Across Industrialized and Non-Industrial Environments”

**This file includes:**

- Supplementary Materials and Methods
- Figure A. Maps of participant villages across a lifestyle gradient in Kenya and Malaysia
- Figure B. Opposing effects of APOE ε4 and APOE ε2 isoforms on cholesterol
- Figure C. Sex-stratified analyses of lipids and innate immune biomarkers for each population
- Figure D. Primary analyses of lipids and innate immune biomarkers for each of the three major Orang Asli subgroups
- Figure E. Genotype, lifestyle, and GxE interaction effects on innate immune biomarkers
- Figure F. Genotype effects on Orang Asli immune gene expression levels
- Figure G. Cholesterol, lifestyle, and cholesterol x lifestyle effects on innate immune biomarkers
- Figure H. Using different age cutoffs to define completed fertility does not impact conclusions
- Figure I. Power to detect genotype effects on fertility
- Figure J. Top causes of death among children in Kenya and Malaysia (2000-2024) from the UN Inter-agency Group for Child Mortality Estimation
- Supplementary References

**The following are provided in a separate file:**

- Table A. Orang Asli participants broken down by self-reported ethnolinguistic group
- Table B. Turkana and Orang Asli participant demographic information
- Table C. Results from linear models testing for lifestyle effects on body fat percentage and body mass index
- Table D. Results from Haldane Exact tests for Hardy-Weinberg equilibrium and frequency of each haplotype per population
- Table E. Results from linear models testing for genotype (number of ε4 or ε2 alleles) and urbanicity effects on cholesterol measurements
- Table F. Results from linear models testing for genotype (coded linearly) and urbanicity effects on cholesterol measurements
- Table G. Percent variance explained by linear model covariates testing for genotype and urbanicity effects on cholesterol measurements
- Table H. Results from linear models testing for genotype (coded linearly) and urbanicity effects on immune biomarkers in Orang Asli
- Table I. Results from linear models testing for genotype (number of ε4 allele) and urbanicity effects on immune biomarkers in Orang Asli
- Table J. Results from linear models testing for genotype (coded linearly) and urbanicity effects on immune biomarkers in Turkana
- Table K. Results from linear models testing for genotype (number of ε4 allele) and urbanicity effects on immune biomarkers in Turkana
- Table L. Results from linear models testing for effects of genotype and proximate lifestyle variables on immune biomarkers in Orang Asli
- Table M. Results from linear models testing for cholesterol and urbanicity effects on immune biomarkers in Orang Asli
- Table N. Results from linear models testing for cholesterol and urbanicity effects on immune biomarkers in Turkana
- Table O. Results from linear models testing for genotype and urbanicity effects on fertility traits in Orang Asli
- Table P. Results from linear models testing for genotype and urbanicity effects on fertility traits in Turkana
- Table Q. Main results (Orang Asli data only) recapitulated using linear mixed effects models

## Supplementary Materials and Methods

### The Turkana of Northwest Kenya

The Turkana are historically a nomadic pastoralist population living in the remote Turkana Basin in northwest Kenya. Traditionally, high amounts of the Turkana diet has been derived from animal products including milk, meat, and blood, though these numbers vary highly depending on the season and geographic area [[1,2]](https://paperpile.com/c/EDtZ6M/kHpg+7InEg). Ongoing infrastructure construction and rapid economic development of Kenya has resulted in the growth of several urban centers in and near traditional Turkana lands, the expansion of small-scale markets, and an increased reliance on agriculture. As a result, most Turkana no longer exclusively practice traditional pastoralism, instead relying on trade, small scale farming, and increasing participation in the market economy. In addition to socioeconomic changes happening within the Turkana region, many Turkana have moved to highly urbanized areas in central Kenya in the last several decades, and now participate fully in the market-economy [[1,3]](https://paperpile.com/c/EDtZ6M/kzAKO+kHpg).

We used data collected by the Turkana Health and Genomics Project (THGP) (March 2018-November 2023) described in Lea et al [[1]](https://paperpile.com/c/EDtZ6M/kHpg). Following meetings with community leaders and members, the THGP project recruited consenting, healthy adults (>18 years) to participate in structured surveys with a research team member who was familiar with the local community and language of the participant (e.g., Turkana, Swahili, English). All community members, regardless of whether they participated in the project, were provided free primary healthcare and consultation from a local nurse working with the project if desired. Individuals who wished to participate took part in structured interviews asking about demography, subsistence and labor practices, material wealth and housing infrastructure, diet, health, and other aspects of lifestyle. Written, informed consent was obtained from all participants after the study goals, sampling procedures, and potential risks were explained to participants in their native language by researchers. Data collection is further described in [[1,4]](https://paperpile.com/c/EDtZ6M/kHpg+eawu).

### The Orang Asli of Peninsular Malaysia

“Orang Asli” refers broadly to the Indigenous peoples of Peninsular Malaysia. The Orang Asli are typically categorized into three broad groups (the Negrito or Semang, Senoi, and Proto-Malay) and 19 culturally distinct ethnolinguistic groups. While these three subgroups are somewhat genetically distinct, the Orang Asli are generally much more genetically similar to each other than to ethnic Malays or other Asian populations [[5]](https://paperpile.com/c/EDtZ6M/4fU8). Collectively, the Orang Asli traditionally practiced varied subsistence histories consisting of foraging, swidden agriculture, trade of rainforest products, or a combination thereof, and these practices were ubiquitous among Orang Asli until the 1950’s and remain common in some areas today [[6,7]](https://paperpile.com/c/EDtZ6M/HbdG+j8Zl). Over the last several decades, Malaysia has undergone one of the fastest rates of urbanization, deforestation, and industrialization worldwide [[8,9]](https://paperpile.com/c/EDtZ6M/GJqI+Tl57), which, combined with government efforts to assimilate the Orang Asli into the national economy, has resulted in Orang Asli experiencing a wide gradient of lifestyles. This ranges from individuals living in remote interior rainforest villages and practicing small-scale, subsistence level lifestyles such as foraging and hunting to Orang Asli villages being surrounded by urban or peri-urban development and individuals participating in the market economy instead of, or in addition to, traditional practices [[7]](https://paperpile.com/c/EDtZ6M/j8Zl).

We used data from the Orang Asli Health and Lifeways Project (OA HeLP) that were collected between March 2020 and October 2024. OA HeLP works in Orang Asli communities and performs similar sampling and data collection as the THGP. OA HeLP also provides free primary healthcare to community members regardless of participation status in collaboration with local hospitals and NGOs. After consent from community leaders and hosting community-wide informational sessions, OA HeLP invited adults to participate in the study. The study aims, procedures, and potential risks were explained to all participants and written, informed consent was obtained from all participants. Additional information about OA HeLP is described in [[10]](https://paperpile.com/c/EDtZ6M/VNe5).

### Urbanicity scale generation

Turkana and Orang Asli live across wide lifestyle gradients, which we have previously described using a location-based “urbanicity” score [[11–14]](https://paperpile.com/c/EDtZ6M/CTOL+bKlE+sUk4+E6C8). Using a location-level scale is advantageous because it allows individuals who might be missing individual-level data to be included in analysis and because it gives a representation of the resources available across the community (e.g., an individual may not own a television but may have access through family or friends). To quantify individuals’ exposure to urban centers and urban, industrialized, and market-integrated infrastructure comparably in both populations, we used a scale that was first proposed by [[15]](https://paperpile.com/c/EDtZ6M/Sglc). This scale was tested in Orang Asli and Turkana in [[11]](https://paperpile.com/c/EDtZ6M/CTOL) and predicted cardiometabolic health better than other measures of urbanicity, acculturation, industrialization, and market-integration. Scale construction is shown below. Population density was estimated from NASA’s Gridded Population of the World resource with a resolution of 2.5 arc-minutes [[16]](https://paperpile.com/c/EDtZ6M/cOQd). The urbanicity score values ranged from 4 - 41.8 in Orang Asli and between 0.67 - 41.4 in Turkana, emphasizing highly comparable lifestyle gradients. We modeled urbanicity score as a continuous variable in our analyses but for the purposes of plotting, we show data from “rural” and “urban” individuals following a natural break in the bimodal distribution of urbanicity in both populations at 25. Urbanicity scores per sampling location are shown in Figure A, plotted with the rnaturalearth package in the R environment using public domain maps from Natural Earth.

| **Population density (estimated number of people per square kilometer)** | **Contribution to scale** |
| --- | --- |
| 0-100 | 0.67 |
| 100-200 | 1.33 |
| 200-300 | 2 |
| 300-400 | 2.67 |
| 400-500 | 3.33 |
| 500-1000 | 4 |
| 1000-2000 | 4.67 |
| 2000-3000 | 5.33 |
| 3000-4000 | 6 |
| 4000-6000 | 6.67 |
| 6000-8000 | 7.33 |
| 8000-10000 | 8 |
| 10000-15000 | 8.67 |
| 15000-20000 | 9.33 |
| >20000 | 10 |
| **Occupation** |  |
| Proportion of the population involved in non-wage labor | 10 - (10 x (proportion)) |
| **Built Environment** |  |
| Proportion of households with flush toilets | 5 x proportion |
| Proportion of households with electricity | 5 x proportion |
| **Communication/market-derived items** |  |
| Proportion of households with mobile phone | 5 x proportion |
| Proportion of households with television | 5 x proportion |
| **Education** |  |
| Proportion of surveyed individuals > 40 years with any level of formal education | 10 x proportion |
| Proportion of surveyed individuals < 40 years with any level of formal education | 10 x proportion |

### Orang Asli accelerometry data collection and processing

OA HeLP study participants were given a triaxial accelerometer (Axivity AX3, Axivity Ltd., UK) to wear for up to 10 days on their non-dominant wrist to measure their physical activity patterns (median = 6 wear days per person). The devices were later collected by a community volunteer and eventually returned to a member of the research team. Accelerometry is purposefully restricted to periods when researchers are not working in the community to ensure that behavior is not altered by the presence of the research team.

Prior to deployment of the accelerometers, the devices were configured to collect data at a sampling frequency of 100 Hz with a dynamic range of 8 g. Raw accelerometry files were downloaded and pre-processed to remove any days recorded after participants wore the devices. We then used the GGIR package v. 3.2.6 in R v. 4.4.2 to process data into 5-second epochs for analysis using default auto-calibration and imputation algorithms [[17,18]](https://paperpile.com/c/EDtZ6M/hbIE+LFiu). Non-wear time was estimated using built-in GGIR algorithms, and we defined a valid day for inclusion as those in which ≥16 hours of wear time were detected. We extracted the Euclidean Norm Minus One metric—a common summary measure of overall activity levels for the day—from the GGIR output. Specifically, this metric is the Euclidean norm (vector magnitude) of the x, y, and z axes (for a tri-axial device) representing raw signals of acceleration, accounting for the effect of gravity by subtracting one gravitational unit.

### Orang Asli gene expression data generation and processing

We measured gene expression levels in peripheral blood mononuclear cells isolated from whole blood for 755 Orang Asli individuals. Gene expression data were generated in June 2025. To do so, approximately 8mL of venous blood was collected from each participant in a CPT tube, from which peripheral blood mononuclear cells (PBMCs) were isolated following the manufacturer’s instructions. PBMCs were preserved in Zymo’s DNA/RNA shield and stored in liquid nitrogen for transport followed by longer-term storage at -80C. RNA was extracted from the PBMCs using Zymo Quick-RNA 96 kits, Zymo Quick-DNA/RNA MagBead kits, or Zymo Quick-RNA MiniPrep Plus kits and were library prepped using the NEBNext Ultra II RNA library prep kit. Samples were molarity-normalized and sequenced on the Illumina NovaSeq X sequencing platform at The Translational Genomics Research Institute (TGen) or the Vanderbilt University Medical Center (VUMC) Vanderbilt Technologies for Advanced Genomics (VANTAGE) core. All samples were sequenced to an average depth of 18,142,612 total reads (range = 3,246,661 - 67,538,791).

We followed standard pipelines for read trimming and removing adapter contamination as described in [[19–21]](https://paperpile.com/c/EDtZ6M/C1zN+92cY+9OnW). Next, reads were mapped to the human reference genome (hg38) using STAR [[20]](https://paperpile.com/c/EDtZ6M/92cY) and gene counts were compiled using HTSeq [[21]](https://paperpile.com/c/EDtZ6M/9OnW). As further quality control, given that our PBMC samples were isolated from whole blood, we calculated the percent of reads mapping to hemoglobin-related genes (specifically HBA1, HBA2, and HBB) and removed any samples with >10% hemoglobin-associated reads. We also excluded any samples with low read counts (<1 million reads), low mapping rates (<70% of reads uniquely mapped to the human genome), or whose sex inferred from the transcriptomic data (i.e., from Y chromosome read counts) did not match their self-reported sex (indicating a potential sample mix up or labeling issue).

### Orang Asli genetic relatedness matrix construction

We performed a set of supplementary analyses in Orang Asli that controlled for pairwise genetic relatedness using linear mixed effects models. To generate these values, we first derived individual-level genotype calls from the mRNAseq reads. To do so, aligned BAM files were processed using a variant-calling pipeline based on GATK (v4.1.4.0) and SAMtools (v1.9) [[22,23]](https://paperpile.com/c/EDtZ6M/L140+tC5I). Briefly, coordinate-sorted BAMs were filtered to retain only autosomal reads and indexed with SAMtools. Duplicate reads were identified and marked using Picard (v2.18.27), and read group information was added to ensure compatibility with downstream GATK tools [[24]](https://paperpile.com/c/EDtZ6M/e9Xn). GATK’s SplitNCigarReads was used to adjust spliced alignments for RNA-seq data. GATK’s base quality score recalibration (BQSR) was performed using known high-confidence SNPs from the 1000 Genomes Project phase 1 as reference sites [[25]](https://paperpile.com/c/EDtZ6M/t18B).

Variant calling was conducted per sample using GATK’s HaplotypeCaller in gVCF mode, followed by variant quality filtering to remove calls with Fisher Strand (FS > 30.0) or low quality-by-depth (QD < 2.0). The resulting per-sample filtered VCFs were zipped, indexed, and merged across all individuals with bcftools (v1.18) [[26]](https://paperpile.com/c/EDtZ6M/lHSG). Only variants with the PASS filter flag were retained for subsequent analyses. The merged multi-sample VCF was further filtered to remove variants with excessive missingness (allele number < 50% of samples), minor allele frequency (MAF) < 1%, or Hardy-Weinberg equilibrium (p<1×10^−6^), using PLINK (v1.9) [[27]](https://paperpile.com/c/EDtZ6M/t3B2). After all filtering steps, high-quality biallelic SNPs were retained for genotype-based quality control. Pairwise kinship coefficients were computed using KING (v2.3.2) to confirm sample independence and identify potential duplicates [[28]](https://paperpile.com/c/EDtZ6M/nZcb). The final high-confidence genotype matrix was used to generate a pairwise genetic relatedness matrix in Plink for downstream linear mixed effects modeling.

### Orang Asli gene expression data analysis

To test whether gene expression levels differed across APOE genotypes, we used the R package EMMREML [[29]](https://paperpile.com/c/EDtZ6M/NrfR) to employ a linear mixed modeling approach. Gene expression counts were first filtered to remove lowly expressed genes (average transcripts per million > 2), normalized using the voomWithQualityWeights function in the R package limma [[30]](https://paperpile.com/c/EDtZ6M/ulKr), and corrected for sequencing batch using lmFit in limma. We modeled the normalized, batch-corrected expression values for 9993 genes using models that included the effects of APOE genotype (linear variable), while controlling for urbanicity, age, sex, percent of lymphocytes and monocytes from complete blood count with differential, and genetic relatedness between individuals. We corrected for multiple hypothesis testing using the qvalue package in R [[31]](https://paperpile.com/c/EDtZ6M/QzvX). We also perform sensitivity analyses to ask whether the effects of genotype on gene expression are sex-dependent by using the described modeling structure within males, within females, and with models with an interaction between sex and genotype. We also ask whether the effects of APOE genotype on gene expression are consistent across the three major Orang Asli subgroups by performing subgroup-specific analyses.

Though we observed a non-uniform distribution of genotype-associated p-values, indicating a likely global effect, we did not find any genes that were significantly associated with genotype at a 10% false discovery rate cutoff. Nevertheless, to identify biological processes that were enriched in genes with the largest APOE genotype effect sizes, we conducted a gene set enrichment analysis using the fgsea package in R [[32]](https://paperpile.com/c/EDtZ6M/fWdL), which preranks genes by their effect size and does not require a set of significantly differentially expressed genes as input.

### Turkana relatedness and population structure

To the best of our knowledge, the THGP dataset contains minimal close relatives (e.g., a single individual per household is invited to participate); this aspect of sampling design, as well as a lack of evidence for major confounding between lifestyle and population structure, has previously been described using genotype array data (Infinium Global Screening Array) [4]. While we unfortunately do not have substantial overlap between the genotyping array data used in Lea et al. and our APOE-genotyped samples and therefore cannot apply linear mixed effects models broadly for our Turkana analyses, we attempted several investigations to understand the potential for confounding from population stratification. First, we reanalyzed the genotyping array data from Lea et al. (n = 994 individuals) to show that the first PC of genetic variation—which explains 19% of the variance—is not associated with the measure of urbanicity used in this study (Pearson’s correlation: 0.04, p = 0.331). PCs 2-4 are however modestly associated (Pearson’s correlation ranging from 0.14 to 0.21, all p<0.05), motivating future work to still strive to include linear mixed effects approaches. Second, we asked whether, across the primary dataset of Turkana samples used in this study, there was a correlation between APOE dosage and urbanicity. We did not find support for such a relationship (Pearson’s correlation = 0.004, p = 0.904). Third, we asked whether any of the top 5 genetic PCs were significantly associated with APOE genotype dosage, focusing on the set of samples that have both APOE and array data (n = 126). All correlation coefficients are non-significant (Pearson’s correlation ranging from 0.03 to 0.13, all p>0.05), though the lack of significance could be due to the limited sample size. Thus, while we cannot exclude the possibility that subtle population structure impacts our analyses, these effects are likely overall small in magnitude.

### Outlier detection and filtering of lipid, innate immune, and fertility datasets

Before modeling our cholesterol, innate immune biomarker, or fertility datasets, we first removed outliers. We did not observe any obvious outliers in the three blood lipids we measured (range of lipid values for Orang Asli: total cholesterol = 100-400, HDL = 20-120, LDL = 29-288; range of lipid values for Turkana: total cholesterol = 99-332, HDL = 19-121, LDL = 1-188). We performed a log2 transformation of immune cell counts, cytokine values, and C-reactive protein and removed values that surpassed an interquartile range (IQR) of 2.

For our menopause analyses, if women reported being menopausal but also reported that they were currently cycling, pregnant, or breast feeding we changed these responses to pre-menopausal. We also excluded observations where menopause occurred prior to the age of 40 or after 62, as menopause occurring outside of this age range may have a different etiology than typical menopause [[33,34]](https://paperpile.com/c/EDtZ6M/90YM+CqWL). In a very large, US representative dataset (NHANES), age at menarche ranged from 8-20 years old and thus we removed entries with age at menarche outside this range in our data (n=7). Following [[35]](https://paperpile.com/c/EDtZ6M/1NZA), we also excluded observations where age at first birth was greater than 30, as this likely reflected extenuating reproductive circumstances. Lastly, we excluded observations where the number of births exceeded the number of pregnancies (n=1) or the number of reproductively mature years would not allow for the number of live births reported (n=3) as these were likely recording errors.

### Power analyses

We estimated power to detect differences in completed fertility between APOE ε4 carriers and non-carriers using a simulation-based framework that mirrors the Poisson generalized linear model (GLM) approach used in the main text. First, we specified the observed proportion of individuals carrying at least one APOE ε4 allele in each population (35.2% in Orang Asli and 46.4% in Turkana) and set the total sample size to a number varying from 50 to 400 individuals. For each sample size, we simulated 1000 replicate datasets in which the number of children for non APOE ε4 carriers was generated from a Poisson distribution with a mean of 6.5 (approximately the average number of children in the completed fertility dataset for both groups). APOE ε4 carriers were then assigned an increased mean of 0.5 children, consistent with previously reported effect sizes [[35]](https://paperpile.com/c/EDtZ6M/1NZA). Each simulated dataset was analyzed using a Poisson GLM with genotype as a predictor, and the proportion of simulations in which the genotype coefficient achieved P < 0.05 was taken as the estimated power for a given sample size. Power curves were generated separately for the Orang Asli and Turkana genotype frequencies to illustrate how population-specific allele frequencies influence detectable effect sizes.

### Childhood mortality and primary pathogens afflicting Turkana and Orang Asli

We sought to provide insight into potential selective pressures for APOE evolution in the Turkana and Orang Asli in two ways. First, we assessed data from the UN Inter-agency Group for Child Mortality Estimation for Kenya and Malaysia on causes and rates of child mortality from 2000-2024, focusing on communicable diseases and malnutrition (i.e., excluding non-communicable diseases and accidents) in infants through 19 years of age. Aggregated mean percent of each cause of death across years is shown in Figure J. We note that these data are at the country-level and not specific to Turkana or Orang Asli. As Turkana and Orang Asli are often under-reported in national or international datasets and may face different diseases due to geography or lifestyle, we performed a survey of the literature on major diseases afflicting each group and, separately, whether APOE genotypes have been associated with susceptibility or resilience to that pathogen/disease in any population. We incorporated both contemporary and historical references to capture major infectious diseases present pre-industrialization, as this should better reflect the pathogen landscape that these groups have been exposed to over timescales pertinent for understanding ε4 persistence than current pressures post-industrialization.

## Supplementary Results

Data from the UN Inter-agency Group for Child Mortality Estimation for Kenya and Malaysia show that the major risk factors for child mortality from communicable sources and malnutrition differ substantially between the two countries. Turkana and Orang Asli are likely under-represented in national or international datasets and therefore we performed a literature search specific to these groups which revealed that many of the most commonly reported diseases in each setting are similar between groups, including malaria, enteric diseases (generally bacterial), and respiratory infections. We also note that many studies noted high incidence of co-infection of multiple pathogens or co-incidence of infection and malnutrition. Additionally, we find that outside of malaria, mechanistic or even associative links between APOE genotype and disease risk/protection remains widely understudied or unknown.

**Table 1:** Pathogens prevalent in Orang Asli or Turkana and associations between APOE genotypes and susceptibility/resilience for infection or other outcomes. Up-arrows (↑) denote protection, down-arrows (↓) denote susceptibility.

| **Disease** | **Population** | **APOE association** | **References on prevalence** | **References on APOE associations** |
| --- | --- | --- | --- | --- |
| Malaria | Orang Asli, Turkana | ε4 ↓  Greater susceptibility to severe infection but better long-term cognitive outcomes post-infection | Baer 1999; Bolton 1968; Bolton 1972; Archibald et al. 1990; Clarke et al. 1996; Shell-Duncan 1995; Little and Leslie 1999 | Wozniak et al. 2004; Lima-Cooper et al. 2023; Rougeron et al. 2013; among others |
| General causes of enteric diseases:  soil-transmitted helminths; protozoan infection including *Blastocystis*, giardias, and *Entamoeba histolytica* (amoebiasis) | Orang Asli, Turkana | Giardia and general enteric disease: ε4 ↑ | Mahmud et al. 2022; Mwangi et al. 2026; Shell-Duncan 1995; Little and Leslie 1999 | Oriá et al. 2005; Oriá et al. 2007 |
| Upper or lower tract respiratory infections | Orang Asli, Turkana | Chlamydia pneumoniae: Results are highly mixed  Many other infection types remain under-studied | Cleary et al. 2021; Shell-Duncan 1995; Little and Leslie 1999 | Balin et al. 2018 |
| Tuberculosis | Orang Asli, Turkana | ε2 ↓ / Unknown | Bolton 1968; Maragia et al. 2025; Little and Leslie 1999 | Wozniak et al. 2009 |
| Dengue | Orang Asli | No direct evidence but hypercholeserolemia can facilitate dengue | George 1987 | Ahmad et al. 2021 |
| Malnourishment and anemia concurrent with infections | Orang Asli, Turkana | ε4 ↑ | Baer 1999; Mwangi et al. 2026; Shell-Duncan 1995 | Smith and Ashford 2023; Oriá et al. 2005; several others |
| Hookworm | Orang Asli | Unknown | Baer 1999 | No direct evidence |
| Rabies | Orang Asli | Unknown | Baer 1999 | No direct evidence |
| Schistosomiasis | Orang Asli | Unknown | Baer 1999 | No direct evidence |
| Scrub typhus | Orang Asli | Unknown | Baer 1999 | No direct evidence |
| Trichuris | Orang Asli | Unknown | Baer 1999 | No direct evidence |
| Yellow Fever, West Nile Virus (*Flaviviruses*) | Turkana | Unknown | Chepkorir et al. 2019 | No direct evidence |
| Measles | Turkana | Unknown | Little and Leslie 1999 | No direct evidence |

## Supplementary Figures

**Figure A. Maps of participant villages across a lifestyle gradient in Kenya and Malaysia**. Panel A shows participant communities associated with the Turkana Health and Genomics Project (map of Kenya) and panel B shows participant communities associated with the Orang Asli Health and Lifeways Project (map of Peninsular Malaysia). Shading denotes the urbanicity score for each location and the size of the circle indicates the number of participants. Maps were made using Natural Earth (public domain; 1:10m country boundaries; https://www.naturalearthdata.com/downloads/10m-cultural-vectors/).

**Figure B. Opposing effects of APOE ε4 and APOE ε2 isoforms on cholesterol.** Forest plot of the effect of APOE (coded as the number of APOE ε4 or APOE ε2 alleles) on cholesterol measures, controlling for age, sex, and urbanicity. Points represent the standardized effect size and bars represent 95% confidence intervals. Positive values indicate positive associations between cholesterol traits and allele number. All tests passed a 5% false discovery rate cutoff.

**Figure C. Sex-stratified analyses of lipids and innate immune biomarkers for each population.** Significant results of sex x genotype or sex x genotype x urbanicity models are denoted with asterisks on the right side of plots B and C. Marginally significant p-value of 0.05 is denoted as well.

**Figure D. Primary analyses of lipids and innate immune biomarkers for each of the three major Orang Asli subgroups.** We focus on innate immune biomarkers that showed genotype or genotype by urbanicity effects. The total number of individuals in each subgroup in our analyses are: Senoi = 423, Proto-Malay = 203, and Negrito = 197.

**Figure E. Genotype, lifestyle, and GxE interaction effects on innate immune biomarkers.** Forest plot of effects of genotype (coded linearly), urbanicity, and genotype x urbanicity from a model including controlling for age and sex. Points represent the standardized effect size and bars represent 95% confidence intervals. Positive values indicate positive associations between immune traits and number of APOE ε4 alleles or increasing urbanicity.

**Figure F.** **Genotype effects on Orang Asli immune gene expression levels.** (A) Distribution of p-values for the linear APOE genotype variable, across all 9993 analyzed genes. P-values were extracted from linear mixed effects models controlling for age, sex, cell type composition, urbanicity, and genetic relatedness. (B) Gene set enrichment analysis scores, indicating degree of enrichment in a given pathway, for GSEA hallmark pathways. Pathways are colored by whether they passed a 5% false discovery rate threshold.

**Figure G.** **Cholesterol, lifestyle, and cholesterol x lifestyle effects on innate immune biomarkers** in (A) Orang Asli and (B) Turkana. Forest plots show the magnitude of lifestyle x cholesterol trait interactions on each immune outcome from a linear model controlling for age and sex and corresponding main effects. Points represent the standardized effect size and bars represent 95% confidence intervals. (C) Shows example plots of relationships with significant interactive effects. Data from Orang Asli are shown in the first two plots in red and the third plot shown in blue is from the Turkana.

**Figure H. Using different age cutoffs to define completed fertility does not impact conclusions.** Model estimates for the interaction between APOE genotype and urbanicity in predicting a woman’s lifetime number of births in our completed fertility datasets from the Orang Asli (A) and Turkana (B). Across all age cutoffs considered, we found no significant interaction between APOE genotype and urbanicity. For each cutoff, sample sizes of women included in the completed fertility dataset and the corresponding p-values for the interaction term are reported.

**Figure I. Power to detect genotype effects on fertility.** We performed 1000 replicate simulations for datasets of varying sizes to estimate our power to detect a difference of 0.5 births between APOE ε4 carriers and non-carriers. Power was estimated as the proportion of simulated true effects detected at p < 0.05 in a generalized linear modeling framework. Power is generally low and has not asymptoted for our sample size range.

**Figure J. Top causes of death among children in Kenya and Malaysia (2000-2024) from the UN Inter-agency Group for Child Mortality Estimation.** Plot shows the mean percent across years 2000-2024 of communicable diseases which are tracked, as well as malnutrition (i.e., not accidents, cancers, etc.). Statistics are available by country and are not specific to Turkana or Orang Asli.

## Supplementary References

1.	[Lea AJ, Martins D, Kamau J, Gurven M, Ayroles JF. Urbanization and market integration have strong, nonlinear effects on cardiometabolic health in the Turkana. Sci Adv. 2020;6. doi:](http://paperpile.com/b/EDtZ6M/kHpg)[10.1126/sciadv.abb1430](http://dx.doi.org/10.1126/sciadv.abb1430)

2.	[Galvin K. Nutritional Ecology of Pastoralists in Dry Tropical Africa. Am J Hum Biol. 1992;4: 209–221.](http://paperpile.com/b/EDtZ6M/7InEg)

3.	[Akall G. Effects of development interventions on pastoral livelihoods in Turkana County, Kenya. Pastoralism. 2021;11: 23.](http://paperpile.com/b/EDtZ6M/kzAKO)

4.	[Lea AJ, Caldas IV, Garske KM, Gerlinger ER, Arroyo JP, Echwa J, et al. Adaptations to water stress and pastoralism in the Turkana of northwest Kenya. Science. 2025;389: 1246–1251.](http://paperpile.com/b/EDtZ6M/eawu)

5.	[Aghakhanian F, Yunus Y, Naidu R, Jinam T, Manica A, Hoh BP, et al. Unravelling the genetic history of Negritos and indigenous populations of Southeast Asia. Genome Biol Evol. 2015;7: 1206–1215.](http://paperpile.com/b/EDtZ6M/4fU8)

6.	[Endicott K. Malaysia’s Original People: Past, Present and Future of the Orang Asli. NUS Press; 2015.](http://paperpile.com/b/EDtZ6M/HbdG)

7.	[Nicholas C, International Work Group for Indigenous Affairs. The Orang Asli and the Contest for Resources: Indigenous Politics, Development, and Identity in Peninsular Malaysia. Copenhagen, Denmark : International Work Group for Indigenous Affairs ; Subang Jaya, Malaysia : Center for Orang Asli Concerns; 2000.](http://paperpile.com/b/EDtZ6M/j8Zl)

8.	[Najib NEM. Forest Cover Change in Peninsular Malaysia Using Satellite Remote Sensing Data. 2015.](http://paperpile.com/b/EDtZ6M/GJqI)

9.	[Kathirithamby-Wells J. Nature and Nation: Forests and Development in Peninsular Malaysia. University of Hawaii Press; 2005.](http://paperpile.com/b/EDtZ6M/Tl57)

10.	[Wallace IJ, Lea AJ, Lim YAL, Chow SKW, Sayed IBM, Ngui R, et al. Orang Asli Health and Lifeways Project (OA HeLP): a cross-sectional cohort study protocol. BMJ Open. 2022;12: e058660.](http://paperpile.com/b/EDtZ6M/VNe5)

11.	[Watowich MM, Arner AM, Wang S, John E, Kahumbu JC, Kinyua P, et al. The built environment is more predictive of cardiometabolic health than other aspects of lifestyle in two rapidly transitioning Indigenous populations. medRxiv. 2024. doi:](http://paperpile.com/b/EDtZ6M/CTOL)[10.1101/2024.08.26.24312234](http://dx.doi.org/10.1101/2024.08.26.24312234)

12.	[Petersen RM, Tennyson R, A/P Tan Boon Huat TBT, Tam KL, Watowich MM, Solhaimi bin Fadzil K, et al. Early life environments shape adult cardiometabolic health during rapid lifestyle change. Evolutionary Biology. bioRxiv; 2025. Available:](http://paperpile.com/b/EDtZ6M/bKlE) <https://www.biorxiv.org/content/10.1101/2025.06.06.658350v1>

13.	[Kraft TS, Wallace IJ, Lim YAL, Tam KL, Huat TBTATB, Tennyson R, et al. Physical activity and cardiometabolic health across an extreme lifestyle gradient. Public and Global Health. medRxiv; 2025. Available:](http://paperpile.com/b/EDtZ6M/sUk4) <https://www.medrxiv.org/content/10.1101/2025.07.11.25331394v1>

14.	[Watowich MM, Brassington L, Longtin A, Wang S, Rossow R, Reinhardt KD, et al. Urbanization exacerbates age-associated declines in cardiometabolic health in Turkana and Orang Asli. Epidemiology. medRxiv; 2025. Available:](http://paperpile.com/b/EDtZ6M/E6C8) <https://www.medrxiv.org/content/10.1101/2025.06.06.25329160v1>

15.	[Novak NL, Allender S, Scarborough P, West D. The development and validation of an urbanicity scale in a multi-country study. BMC Public Health. 2012;12: 530.](http://paperpile.com/b/EDtZ6M/Sglc)

16.	[Center For International Earth Science Information Network-CIESIN-Columbia University. Gridded population of the world, version 4 (GPWv4): Population count, revision 11. Palisades, NY: NASA Socioeconomic Data and Applications Center (SEDAC); 2018. doi:](http://paperpile.com/b/EDtZ6M/cOQd)[10.7927/H4JW8BX5](http://dx.doi.org/10.7927/H4JW8BX5)

17.	[Migueles JH, Rowlands AV, Huber F, Sabia S, van Hees VT. GGIR: A research community–driven open source R package for generating physical activity and sleep outcomes from multi-day raw accelerometer data. J Meas Phys Behav. 2019;2: 188–196.](http://paperpile.com/b/EDtZ6M/hbIE)

18.	[van Hees V, Migueles JH. GGIR. Zenodo; 2025. doi:](http://paperpile.com/b/EDtZ6M/LFiu)[10.5281/ZENODO.1051064](http://dx.doi.org/10.5281/ZENODO.1051064)

19.	[Martin M. Cutadapt removes adapter sequences from high-throughput sequencing reads. EMBnet J. 2011;17: 10.](http://paperpile.com/b/EDtZ6M/C1zN)

20.	[Dobin A, Davis CA, Schlesinger F, Drenkow J, Zaleski C, Jha S, et al. STAR: ultrafast universal RNA-seq aligner. Bioinformatics. 2013;29: 15–21.](http://paperpile.com/b/EDtZ6M/92cY)

21.	[Putri GH, Anders S, Pyl PT, Pimanda JE, Zanini F. Analysing high-throughput sequencing data in Python with HTSeq 2.0. Bioinformatics. 2022;38: 2943–2945.](http://paperpile.com/b/EDtZ6M/9OnW)

22.	[Van der Auwera GA, Carneiro MO, Hartl C, Poplin R, Del Angel G, Levy-Moonshine A, et al. From FastQ data to high confidence variant calls: the Genome Analysis Toolkit best practices pipeline. Curr Protoc Bioinformatics. 2013;43: 11.10.1–11.10.33.](http://paperpile.com/b/EDtZ6M/L140)

23.	[Li H, Handsaker B, Wysoker A, Fennell T, Ruan J, Homer N, et al. The Sequence Alignment/Map format and SAMtools. Bioinformatics. 2009;25: 2078–2079.](http://paperpile.com/b/EDtZ6M/tC5I)

24.	[Picard. [cited 22 Dec 2025]. Available:](http://paperpile.com/b/EDtZ6M/e9Xn) <http://broadinstitute.github.io/picard/>

25.	[1000 Genomes Project Consortium, Auton A, Brooks LD, Durbin RM, Garrison EP, Kang HM, et al. A global reference for human genetic variation. Nature. 2015;526: 68–74.](http://paperpile.com/b/EDtZ6M/t18B)

26.	[Danecek P, Bonfield JK, Liddle J, Marshall J, Ohan V, Pollard MO, et al. Twelve years of SAMtools and BCFtools. Gigascience. 2021;10. doi:](http://paperpile.com/b/EDtZ6M/lHSG)[10.1093/gigascience/giab008](http://dx.doi.org/10.1093/gigascience/giab008)

27.	[Purcell S, Neale B, Todd-Brown K, Thomas L, Ferreira MAR, Bender D, et al. PLINK: a tool set for whole-genome association and population-based linkage analyses. Am J Hum Genet. 2007;81: 559–575.](http://paperpile.com/b/EDtZ6M/t3B2)

28.	[Manichaikul A, Mychaleckyj JC, Rich SS, Daly K, Sale M, Chen WM. Robust relationship inference in genome-wide association studies. Bioinformatics. 2010;26: 2867–2873.](http://paperpile.com/b/EDtZ6M/nZcb)

29.	[Kang HM, Zaitlen NA, Wade CM, Kirby A, Heckerman D, Daly MJ, et al. Efficient control of population structure in model organism association mapping. Genetics. 2008;178: 1709–1723.](http://paperpile.com/b/EDtZ6M/NrfR)

30.	[Law CW, Chen Y, Shi W, Smyth GK. voom: Precision weights unlock linear model analysis tools for RNA-seq read counts. Genome Biol. 2014;15: R29.](http://paperpile.com/b/EDtZ6M/ulKr)

31.	[Dabney A, Storey JD. qvalue: Q-value estimation for false discovery rate control. R package version 1.43.0. Patent. 2015.](http://paperpile.com/b/EDtZ6M/QzvX)

32.	[Korotkevich G, Sukhov V, Budin N, Shpak B, Artyomov MN, Sergushichev A. Fast gene set enrichment analysis. bioRxiv. bioRxiv; 2016. doi:](http://paperpile.com/b/EDtZ6M/fWdL)[10.1101/060012](http://dx.doi.org/10.1101/060012)

33.	[Murray A, Bennett CE, Perry JRB, Weedon MN, Jacobs PA, Morris DH, et al. Common genetic variants are significant risk factors for early menopause: results from the Breakthrough Generations Study. Hum Mol Genet. 2011;20: 186–192.](http://paperpile.com/b/EDtZ6M/90YM)

34.	[Costanian C, McCague H, Tamim H. Age at natural menopause and its associated factors in Canada: cross-sectional analyses from the Canadian Longitudinal Study on Aging. Menopause. 2018;25: 265–272.](http://paperpile.com/b/EDtZ6M/CqWL)

35.	[Trumble BC, Charifson M, Kraft T, Garcia AR, Cummings DK, Hooper P, et al. Apolipoprotein-ε4 is associated with higher fecundity in a natural fertility population. Sci Adv. 2023;9: eade9797.](http://paperpile.com/b/EDtZ6M/1NZA)

## References in Literature Review

Ahmad RI, Nor FM, Mui WS, Abdul Rahman TH. The role of apolipoproteins in dengue infection: A review. IIUM Med J Malays. 2021;20(2).

Archibald CP, Mak JW, Mathias RG, Selvajothi S. Antibodies to Plasmodium falciparum in an indigenous population from a malaria endemic area of Malaysia. Acta Trop. 1990;48(2):149–157.

Baer AS. Health, Disease, and Survival: A Biomedical and Genetic Analysis of the Orang Asli of Malaysia. Center for Orang Asli Concerns; 1999.

Balin BJ, Hammond CJ, Little CS, Hingley ST, Al-Atrache Z, Appelt DM, et al. Chlamydia pneumoniae: An etiologic agent for late-onset dementia. Front Aging Neurosci. 2018;10:302.

Bolton JM. Medical services to the aborigines in West Malaysia. Br Med J. 1968;2(5608):818–823.

Bolton J. The control of malaria among the Orang Asli in West Malaysia. Malays J Med. 1972;27(1):10–19.

Clarke D, Ouma JO, Kenny V, McCabe R, Rapuoda B, Watkins WM. A malariometric survey in Turkana District, Kenya: Chemosensitivity in vivo of Plasmodium falciparum infections and identity of the vector. Trans R Soc Trop Med Hyg. 1996;90(3):253–257.

Cleary DW, Morris DE, Anderson RA, et al. The upper respiratory tract microbiome of indigenous Orang Asli in north-eastern Peninsular Malaysia. NPJ Biofilms Microbiomes. 2021;7:1.

Chepkorir E, Tchouassi DP, Konongoi SL, et al. Serological evidence of Flavivirus circulation in human populations in Northern Kenya: An assessment of disease risk 2016–2017. Virol J. 2019;16:65.

Gaire BP, Koronyo Y, Vit JP, et al. Identification of Chlamydia pneumoniae and NLRP3 inflammasome activation in Alzheimer’s disease retina. Nat Commun. 2026;17:771.

George R. Dengue haemorrhagic fever in Malaysia: A review. Southeast Asian J Trop Med Public Health. 1987;18(3):278–283.

Gilman RH, Davis C, Fitzgerald F. Heavy Trichuris infection and amoebic dysentery in Orang Asli children: A comparison of the two diseases. Trans R Soc Trop Med Hyg. 1976;70(4):313–316.

Lima-Cooper G, Ouma BJ, Datta D, et al. Apolipoprotein-E4: Risk of severe malaria and mortality and cognitive impairment in pediatric cerebral malaria. Pediatr Res. 2024;96:89–96.

Little MA, Leslie PW. Turkana Herders of the Dry Savanna: Ecology and Biobehavioral Response of Nomads to an Uncertain Environment. Oxford: Oxford University Press; 1999.

Mahmud MH, Baharudin UM, Md Isa Z. Diseases among Orang Asli community in Malaysia: A systematic review. BMC Public Health. 2022;22(1):2090.

Maragia JM, Kiplagat J, Dickens O, et al. Predictors of pediatric tuberculosis disease in a pastoralist community, Turkana County, Kenya: A case-control study. BMC Infect Dis. 2025;25:1317.

Mwangi B, Flax VL, Thuita F, Miller JD, Lutter C, Amugsi D, et al. Prevalence and risk factors of diarrhea among young children in Kenya's drylands: A longitudinal study. PLOS Glob Public Health. 2026;6(1):e0003998.

Oriá RB, Patrick PD, Zhang H, Lorntz B, de Castro Costa CM, Brito GA, et al. APOE4 protects the cognitive development in children with heavy diarrhea burdens in Northeast Brazil. Pediatr Res. 2005;57:310–316.

Oriá RB, Patrick PD, Blackman JA, Lima AA, Guerrant RL. Role of apolipoprotein E4 in protecting children against early childhood diarrhea outcomes and implications for later development. Med Hypotheses. 2007;68(5):1099–1107.

Oriá RB, Smith CJ, Ashford JW, Vitek MP, Guerrant RL. Pros and cons of APOE4 homozygosity and effects on neuroplasticity, malnutrition, and infections in early life adversity, Alzheimer’s disease, and Alzheimer’s prevention. J Alzheimers Dis. 2024;100(S1):S179–S185.

Rougeron V, Woods CM, Tiedje KE, Bodeau-Livinec F, Migot-Nabias F, Deloron P, et al. Epistatic interactions between apolipoprotein E and hemoglobin S genes in regulation of malaria parasitemia. PLoS One. 2013;8(10):e76924.

Shell-Duncan B. Impact of seasonal variation in food availability and disease stress on the health status of nomadic Turkana children: A longitudinal analysis of morbidity, immunity, and nutritional status. Am J Hum Biol. 1995;7:339–355.

Smith CJ, Ashford JW. Apolipoprotein ε4-associated protection against pediatric enteric infections is a survival advantage in pre-industrial populations. J Alzheimers Dis. 2023;93(3):907–918.

Wozniak MA, Riley EM, Itzhaki RF. Apolipoprotein E polymorphisms and risk of malaria. J Med Genet. 2004;41:145–146.

Wozniak MA, Maude RJ, Innes JA, Hawkey PM, Itzhaki RF. Apolipoprotein E-ε2 confers risk of pulmonary tuberculosis in women from the Indian subcontinent: A preliminary study. J Infect. 2009;59(3):219–222.
